# 309 metagenome assembled microbial genomes from deep sediment samples in the Gulfs of Kathiawar Peninsula

**DOI:** 10.1038/s41597-021-00957-0

**Published:** 2021-07-28

**Authors:** Neelam M. Nathani, Kaushambee J. Dave, Priyanka P. Vatsa, Mayur S. Mahajan, Parth Sharma, Chandrashekar Mootapally

**Affiliations:** 1 Department of Life Sciences, Maharaja Krishnakumarsinhji Bhavnagar University, Bhavnagar, 364001 Gujarat India; 2 Department of Molecular Cell Biology and Immunology, University of Tübingen, Geschwister-Scholl-Platz, 72074 Tübingen, Germany; 3 Department of Biotechnology, National Institute of Pharmaceutical Education and Research (NIPER), Gandhinagar, 382355 Gujarat India; 4 Microbiology Division, Regional Centre, Lokhandwala Road, Four Bungalows, Andheri (West), CSIR – National Institute of Oceanography (CSIR-NIO), Mumbai, 400053 Maharashtra India; 5 Department of Marine Science, Maharaja Krishnakumarsinhji Bhavnagar University, Bhavnagar, 364001 Gujarat India

**Keywords:** Environmental biotechnology, Biodiversity

## Abstract

Prokaryoplankton genomes from the deep marine sediments are less explored compared to shallow shore sediments. The Gulfs of Kathiawar peninsula experience varied currents and inputs from different on-shore activities. Any perturbations would directly influence the microbiome and their normal homeostasis. Advancements in reconstructing genomes from metagenomes allows us to understand the role of individual unculturable microbes in ecological niches like the Gulf sediments. Here, we report 309 bacterial and archaeal genomes assembled from metagenomics data of deep sediments from sites in the Gulf of Khambhat and Gulf of Kutch as well as a sample from the Arabian Sea. Phylogenomics classified them into 5 archaeal and 18 bacterial phyla. The genomes will facilitate understanding of the physiology, adaptation and impact of on-shore anthropogenic activities on the deep sediment microbes.

## Abstract (summary)

**Table:** 

Measurement(s)	marine metagenome • sequence_assembly
Technology Type(s)	DNA sequencing • sequence assembly process • Binning clustering method
Factor Type(s)	gulf
Sample Characteristic - Environment	marine biome • deep marine sediment
Sample Characteristic - Location	Gulf of Kutch • Gulf of Khambhat • Arabian Sea

Machine-accessible metadata file describing the reported data: 10.6084/m9.figshare.14445387

## Background & Summary

Marine microbiome is considered as the largest environment on earth which has many secrets concealed into it^1,2^. Many marine microbes play a key role in biogeochemical cycles. However, high proportions of microbes remain uncultured *in vitro*^3^ and so instead of analysing the microbes individually, cultivation-independent genome-level characterization methods notably single-cell genomics and metagenomics are frequently being applied for microbiome analysis^4^. Amplicon sequencing based cultivation-independent studies are enriching the microbial diversity knowledge of various hitherto less studied environmental niche, specifically within the marine resources. However, amplicon analysis is just a preliminary step in metagenomics as it focuses only on one gene for the community diversity assessment.

With the view of studying the marine microbial community for determination of its composition in terms of diversity as well as function, whole metagenomics has become the preferred approach. Recently, it has been realized that the actual understanding of metagenomics data can be obtained by individual genome binning, which eventually also enhances the microbial genome database^5^. This requires use of various complex computational algorithms including those relying on previous data findings viz., the supervised classifiers and the unsupervised classifiers that rely on sequence specific features like the GC content, k-mer frequency and coverage estimation for binning the genomes. Most of the recently developed tools for binning include a combined approach of both the algorithms^6^. Binning aids in revealing the link between the potential functional genes in a given microbiome to its taxonomy.

The unique properties of the Gulfs of Kathiawar Peninsula like extreme tidal variations, different sediment texture and physicochemical variations make them an ideal place for studying the microbial diversity. Varied onshore anthropogenic activities may have imparted unique features to the microflora of the Gulfs. Study of microbial diversity and functions in the mentioned Gulfs have largely been focused on cultivation based approaches and very few molecular studies have been conducted on the shore sediments. Additionally, the presence of several on-shore industries like fertilizer, chemicals, oil refineries, power plants and ASSBRY (Alang Ship Breaking Yard) may have also influenced the deeper sediment microbiome leading to their variable gene profile^7^. Our previous insights into the pelagic sediment resistome profile by metagenomics approach have shown that the deeper sediments, earlier thought to be primeval are actually hosting microbes with a concerning number of resistance genes^7,8^. This acted as a propeller to the present study wherein we tried to look deeper into the metagenomics data of the samples collected from the Gulfs of Kathiawar Peninsula and a sample from the Arabian Sea by sorting individual prokaryoplankton genomes from the data using the binning approach.

We successfully reconstructed 309 Metagenome Assembled Genomes (MAGs) from the nine sediment metagenomics sequences (Table 1) from Gulf of Khambhat (GOC), Gulf of Kutch (GOK) and Arabian Sea (A) by differential coverage approach and considering the GC percent and tetranucleotide frequencies. Out of the 309 MAGs, 39 were archaeal genomes (Online-only Table 1) and 270 were bacterial genomes (Online-only Table 2). Seventy-one were high quality drafts with a completeness of ≥90% and contamination <10%, 120 were medium quality (completeness: 70–90%, contamination: <10%) and the remaining 118 were draft genomes with a final completeness of >50%. The distribution of the bins as per the MIMAG quality standards^9^ is described in Table 2. To the best of our knowledge, this is the first report of multiple MAGs from the studied sites.

**Table 1:** Data availability of metagenomic sequence reads used to compute the pooled assembly and further MAGs.

Metagenome	Bioproject ID (EBI and MGnify)	EBI accession	MGnify accession	Pooled assembly accession (NCBI)
A	PRJEB26614/ERP108616/MGYS00002380	ERS2466930	MGYA00475148	GCA_012974985.1/JABAOH000000000 [Bioproject: PRJNA623070, Sample: GOC_A_pooled]
GOCS1		ERS2466926	MGYA00166412	
GOCS2		ERS2466927	MGYA00166413	
GOCS3		ERS2466928	MGYA00475145	
GOCS4		ERS2466929	MGYA00166411	
GOKS1	PRJEB26615/ERP108617/MGYS00002379	ERS2466931	MGYA00166409	GCA_012974765.1/JABAHS000000000 [Bioproject: PRJNA622989, Sample: GOKZ_pooled]
GOKS2		ERS2466932	MGYA00166408	
GOKS3		ERS2466933	MGYA00166410	
GOKS4		ERS2466934	MGYA00475146

**Table 2:** Details of the number of MAGs from this study passing MIMAG quality standards.

Quality standard	Quality	Criteria used for quality standard	# MAGs meeting quality standard	Remark
**Current manuscript**	High	**Criteria 1:** > = 90% completeness **Criteria 2:** < = 10% contamination **Criteria 3:** > = 5 kb N50	71	—
	Medium	**Criteria 1:** 70–90% completeness **Criteria 2:** < = 10% contamination **Criteria 3:** Failing high quality standard	120	—
	Low	Failing medium quality standard	118	—
**Minimum Information about a Metagenome-Assembled Genome (MIMAG) Bowers*et al****.***2017**	High	**Criteria 1:** > = 90% completeness **Criteria 2**: < = 5% contamination **Criteria 3:** genes for > = 18/20 tRNAs **Criteria 4:** genes for 5 S, 16 S, 23 S rRNAs	46	46 MAGs fullfilled the criteria 1 to 3, however, 37 from these MAGs lacked one or more rRNA gene/s (criteria 4)
	Medium	**Criteria 1:** > = 50% completeness **Criteria 2: <** = 10% contamination **Criteria 3:** Failing high quality standard	186	—
	Low	Failing medium quality standard	77	—

**Fig. 1:**
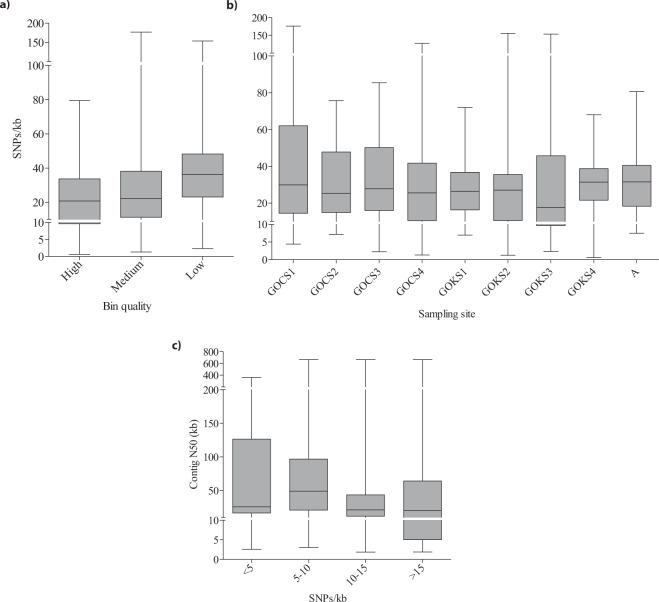
Single nucleotide polymorphisms (SNPs) were called for the MAGs reported here and compared with (**a**) quality, (**b**) sample site and (**c**) N50. The values were plotted as box plot with Min/Max whiskers and line in the middle corresponding to the mean value.

**Fig. 2:**
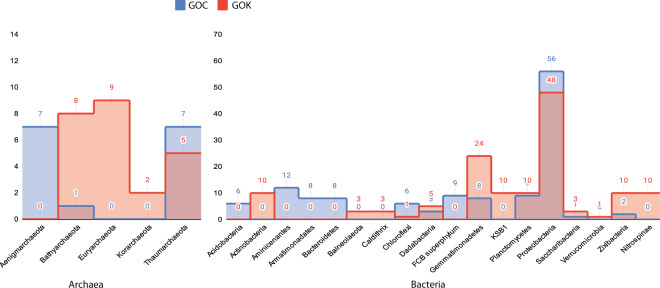
The distribution of MAGs across archaeal and bacterial phyla in the studied sites. Four MAGs that were classified only up to the domain level have not been depicted here.

**Fig. 3:**
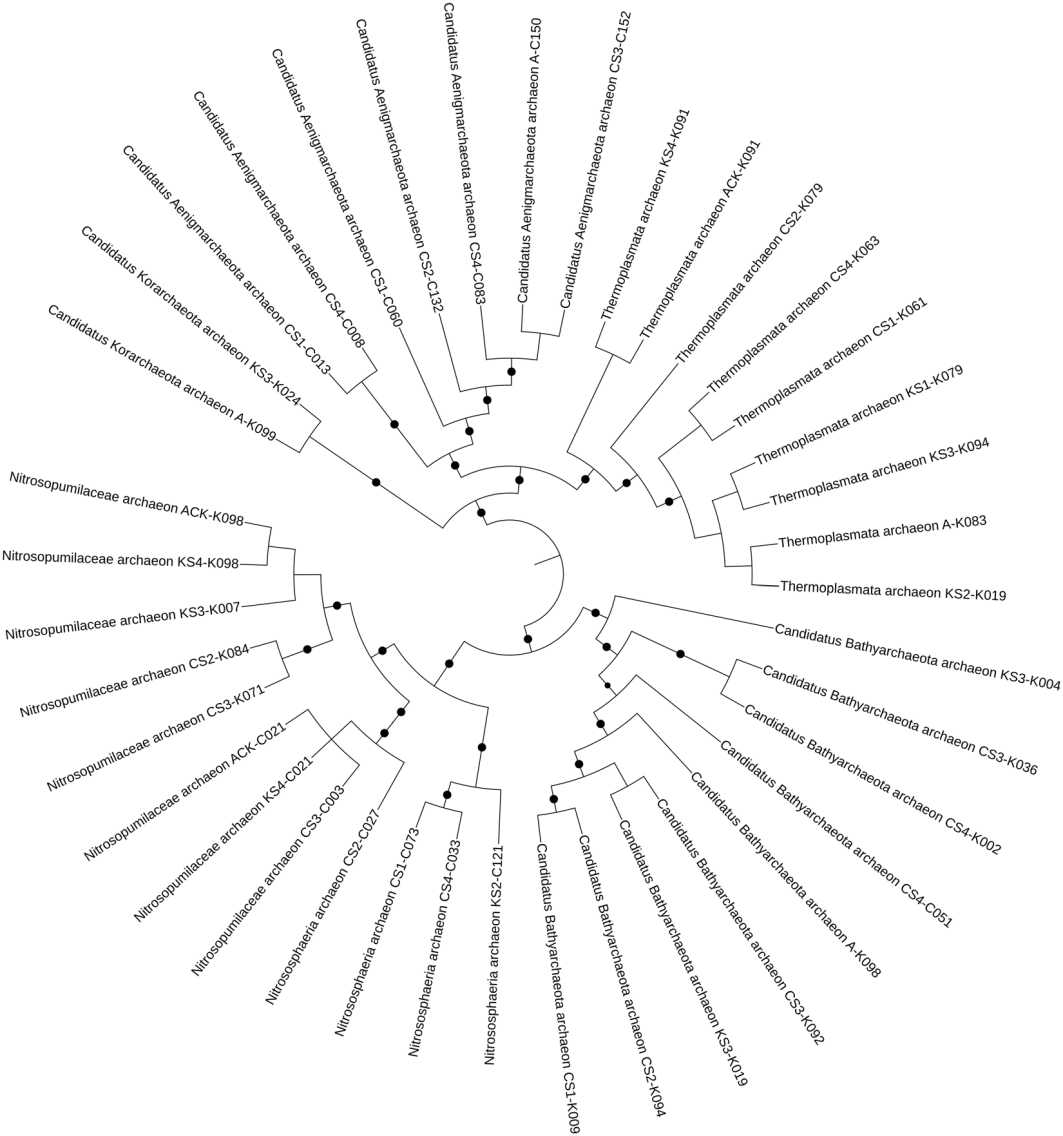
Phylogenetic tree of the archaeal MAGs. Validity of the tree is indicated by filled black circles, size indicates bootstrap between 80 to 100%. The tree was rooted with the Aenigmarchaeota of the DPANN superphylum^4^.

Single nucleotide polymorphisms were correlated to quality of bins to understand the influence of strain heterogeneity on the fragmentation of the MAGs (Fig. 1). Phylogenomic analysis revealed that the archaeal populations were quite different in two Gulfs, with GOC bins (n = 15) encompassing 3 major phyla: Thaumarchaeota and Aenigmarchaeota from the DPANN superphylum andBathyarchaeota. The GOK genomes (n = 24) were falling under the Bathyarchaeota, Thaumarchaeota, Euryarchaeota and the Korarchaeota phyla (Figs. 2 and 3). Based on the community profile assessment of the samples by considering all the reads, the above mentioned archaeal phyla represented <3% of the total microbial population at each sample site. Majority of the phyla were those reported earlier in the marine and estuarine environments, with most having few or no cultured representatives^10,11^. The observed genomes like Thaumarchaeota have been reported to be nitrifiers in the sediment niche, thus, the insights into their gene content will provide details on the functional significance of the archaea in the respective sample site. Genomes from Thaumarchaeota were recovered from both the sites (Fig. 2). Nevertheless, the difference in the populations observed in two Gulfs can also be studied based on the predicted roles of the genomes and correlation with the niche properties.

**Fig. 4:**
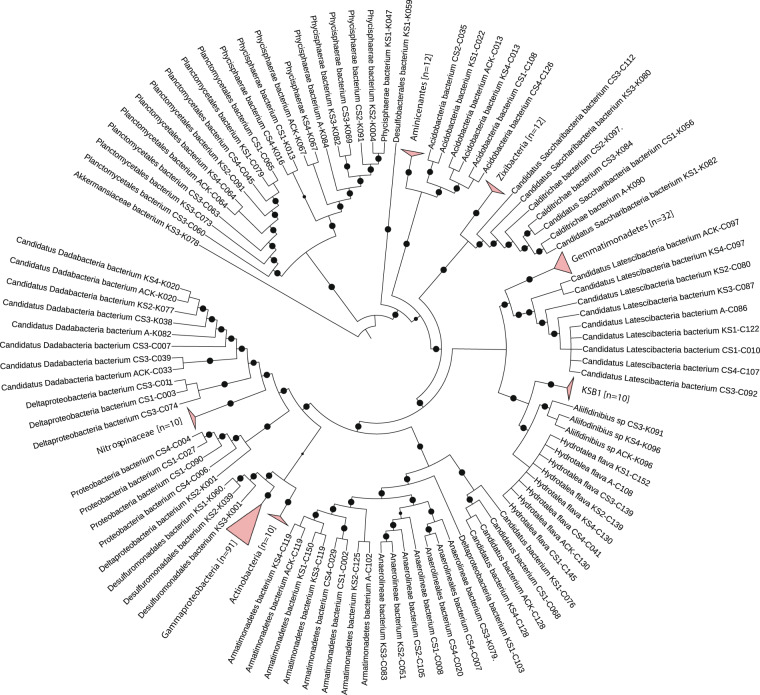
Bacterial clades having ≥10 MAGs classified as the same level are collapsed and represented by triangles, size of which is proportional to the number of genomes collapsed in the taxa level which is also mentioned in the parentheses. Validity of the tree is indicated by filled black circles, size indicates bootstrap between 80 to 100%. The *Akkermansiaceae* (phylum: *Verrucomicrobia*) was arbitrarily taken as the root, the tree may be considered as unrooted^34^.

Among the bacterial members, five phyla were commonly observed between the Gulfs viz., *Proteobacteria, Zixibacteria, Gemmatimonadetes, Dadabacteria* and *Planctomycetes* (Figs. 2 and 4). Among the common bacterial phyla, *Proteobacteria* majorly comprised of Gammaproteobacteria members which are the most abundant reported bacteria in the marine sediments and have been reported to perform versatile roles including metabolite production, hydrocarbon degradation, acetate assimilation and many more^12,13^. *Zixibacteria* and *Dadabacteria* MAGs have been reported from marine environments as an evolutionary phyla and these have been observed to play role in the nutrient cycling of the niche^14,15^. Apart from these, few genomes in GOC encompassed *Bacteroidetes*, FCB superphylum, *Armatimonadates, Acidobacteria, Chloroflexi* and *Aminicenantes* phyla; while those in GOK were falling under *Actinobacteria*, KSB1, *Saccharibacteria* (TM7), *Nitrospinae, Caldithrix*, *Verrucomicrobia* and *Balneolaeota*. Species belonging to *Nitrospinae* are reported to be exclusively abundant in marine niche, where they play a role in nitrite oxidization, as well as these are ubiquitously observed in sites demanding thermoprotection^16,17^. Community profiling of the samples by considering all the reads revealed that the MAGs identified within *Proteobacteria* (>40%) and *Chloroflexi* (~15%) phyla represented a substantial population, while rest of the MAGs corresponded to 0.01% to 5% of the total microbial community at each sample site (details in Supplementary Table 1a and b).

The genomic bins described here would prominently enhance the repertoire of microbial genomic information from the Gulfs of Kathiawar Peninsula. It will also provide the insights for better understanding the effects of on-shore activities on the microbiome of deeper sediment in the Gulfs. In the long term, the data will fortify further applications of the genomic information for 1) understanding the microbes involved in the marine nutrient cycling, 2) open gates for bioprospection of novel thermophilic and halophilic enzymes and 3) allow understanding of microbial-host and microbial-niche interactions as the phylum distribution reflects the variability across the 2 Gulfs under study.

## Methods

### Sample collection and whole metagenome sequencing

The sediment samples were collected and sequenced for whole metagenomics using Illumina HiSeq platform as described earlier^7,8^. In brief, one-meter-long sediment cores were collected from 9 locations across the 2 Gulfs, Gujarat state and open Arabian Sea by sailing through boats. The cores were maintained in cold storage and processed by cutting into halves without disturbing the sediments. 10 cm of sediment from top, middle and bottom of the core each was distributed into 3 sterile 50 ml collection containers. They were further used for assessment of physicochemical properties, metagenomic DNA isolation and culturing purpose. DNA was isolated in multiplicates to reach desired quantity using the MoBio Power Soil DNA isolation Kit (Qiagen, Germany). The DNA from each core section was pooled in equimolar concentrations for whole metagenomics sequencing using HiSeq 4000 (Macrogen Inc., Korea). No internal reference or control was used during the sequencing. The sequences were quality filtered for adaptor removal and a minimum quality score of 20.

### Metagenomics assembly

The quality filtered reads of four GOK samples were used for pooled assembly using CLC Genomics Workbench v11.0 with default parameters except a k-mer size of 31 and a minimum contig length of 1 Kb, which resulted into 478 Mb of assembled data. Similarly, the four samples of GOC along with the A sample were included in another set of pooled assembly of 779 Mb. The raw reads from each of the individual nine samples were mapped against the two assemblies for coverage estimation using CLC Genomics Workbench. The coverage and the BAM files were obtained for further binning process.

### Genome binning

Metagenome assembled genomes (MAGs) were binned from both the assemblies using Maxbin v2.0^6^ using the full reference marker set of genes for bacteria and archaea. More than 900 bins were initially obtained from the pooled datasets. The quality of bins was checked by CheckM v1.1.0 using the lineage-specific workflow and the bins were assessed based on its completeness and contamination values^18^. Contigs with outlier values for the GC percentage and tetranucleotide frequency were removed from the bins for lowering the contamination levels. The assessed bins were further refined using RefineM v0.0.22 by individual genomic properties, taxonomy and SSU based approaches. Further, the individual output of the genomic properties was used as input for the other methods, viz., output bins from the genomic property refined program was further filtered by taxonomic method and so on. The refined bins were re-assessed using CheckM v1.1.0^18^. It was observed that the refinement using the genomic properties which screens for any outlier contigs/scaffoled in a MAG in terms of GC percent, tetranuceotide frequency and coverage did improve the bins in terms of their completeness. While, the taxa based refinement gave an overall improvement of ~2% for few bins and a reduction in the contamination by removal of duplicate or miss-assigned single copy gene encoding contigs. SSU based refinement had no major impact on the MAGs in the study. The bins were then sorted into high quality, medium quality, draft and/or low quality genomes. Out of >900 bins, 309 bins that were falling up to the draft genome category were checked for taxonomic classification using the GTDB-tk v0.3.3 classifier^19^. However, for final submission as suggested by NCBI team, few of the NCBI taxonomic synonyms from GTDB-tk classification were considered. The strain names in the nomenclature were assigned as “sample number of the mapped reads – pooled assembly against which the reads were mapped followed by the number of bin from the mapped sample”, as an example for the strain **CS3**-**K071**, CS3 indicates Gulf of Khambhat/**C**ambay **S**ample **3** which was mapped against the pooled assembly of **K**utch samples and 071 is the bin number from the total bins generated from this mapping. The bins were also submitted to RAST v2.0 for annotation and the number of Protein Encoding Genes (PEGs) for each MAG were inferred from the same for preliminary functional assessment prior to NCBI submission^20^.

### SNP estimation of the MAGs

SNPs were called for each MAG (n = 275, bins generated from all nine samples as a pool were omitted from SNP analysis) (Supplementary Table 1a and b) to assess their genetic diversity as described earlier^21^. For the same, a database of MAGs from each of the nine samples was computed using Bowtie2 v.2.3.4.1^22^ for aligning the respective metagenomic reads. The SNP/Kbps were compared with the quality, sample site and N50 and of the MAGs. All the plots were computed using GraphPad Prism v8.4.1 for Windows^23^.

### Phylogenomic tree construction

The archaeal and bacterial trees were inferred using the insert genome set into species tree app in the Kbase^24^. The annotated bins from NCBI were uploaded as GenBank file and a genome set was prepared using the app Batch Create Genome Set v1.2.0 along with one genome from the database (as default parameter was to take minimum one reference genome). The tree was computed by the alignment of a pre-decided subset of COG (Clusters of Orthologous Groups) domains using FastTree v. 2.1.10^25^, by maximum likelihood phylogeny. The tree was further annotated by iTOL v5.0^26^. The reference genome was hidden during visualization, keeping only the MAGs under the study.

## Data Records

The raw metagenomics reads and their corresponding pooled assemblies are available from EBI and NCBI^27,28^, respectively as detailed in Table 1. The sample-wise metagenome assemblies and pooled assemblies (GOC-A and GOK) are available under the Bioproject Id as mentioned in Table 1^29,30^. The 309 assembled genome sequences and their functional annotations are available from NCBI database^31,32^ via biosample and genome accession numbers as detailed in Online-only Tables 1 and 2. The tree files corresponding to the figures and with reference genomes can be accessed throughfigshare^33^.

## Technical Validation

The quality of MAGs was assessed using CheckM to validate the completeness and contamination of the bins. The genomes were also manually assessed at each point for similar bins by considering the parameters like GC, genome statistics and the number of genes.

## Supplementary information

Supplementary Table 1

## Online-only Tables

**Online-only Table 1:** Genomic properties of the 39 archaeal MAGs reported in the study.

Bin taxonomy (NCBI)	GTDB taxonomy	Size (bp)	No. of contigs	N50	GC (%)	No. of PEGs (RAST)	Genome coverage	Completeness (%)	Contamination (%)	Genome accession (NCBI)	Biosample accession
**GOC**											
Nitrosopumilaceae archaeon CS3-C003	Nitrosopumilaceae archaeon CS3-C003	1474557	74	58313	34.6	2006	528x	97.1	1.9	WVYP00000000	SAMN13684423
Candidatus Aenigmarchaeota archaeon CS4-C008	Candidatus Aenigmarchaeia archaeon CS4-C008	907828	88	30707	31.5	1169	857.6x	72.0	7.5	WVZI00000000	SAMN13684442
Candidatus Aenigmarchaeota archaeon CS1-C013	Candidatus Aenigmarchaeia archaeon CS1-C013	1009083	94	33031	30.9	1326	771.5x	80.5	1.9	WVXN00000000	SAMN13684395
Nitrosopumilaceae archaeon ACK-C021	Nitrosopumilaceae archaeon ACK-C021	1370394	42	59249	34.7	1825	568.1x	96.1	1.9	WVWY00000000	SAMN13684380
Nitrosopumilaceae archaeon KS4-C021	Nitrosopumilaceae archaeon KS4-C021	1370394	42	59249	34.7	1825	568.1x	96.1	1.9	WWBP00000000	SAMN13684501
Nitrososphaeria archaeon CS2-C027	Nitrososphaeria archaeon CS2-C027	984039	24	69356	34.8	1288	791.2x	83.5	0.0	WVYF00000000	SAMN13684413
Nitrososphaeria archaeon CS4-C033	Nitrososphaeria archaeon CS4-C033	2031644	246	19062	47.5	2499	383.2x	83.0	7.3	WVZR00000000	SAMN13684451
Candidatus Aenigmarchaeota archaeon CS1-C060	Candidatus Aenigmarchaeia archaeon CS1-C060	2880460	781	5215	42.2	4296	270.3x	75.5	9.4	WVXR00000000	SAMN13684399
Nitrososphaeria archaeon CS1-C073	Nitrososphaeria archaeon CS1-C073	2043576	321	15271	46.9	2622	381x	71.7	4.4	WVXW00000000	SAMN13684404
Candidatus Aenigmarchaeota archaeon CS4-C083	Candidatus Aenigmarchaeia archaeon CS4-C083	834329	58	48980	46.0	974	933.1x	73.7	2.1	WVZW00000000	SAMN13684456
Nitrososphaeria archaeon KS2-C121	Nitrososphaeria archaeon KS2-C121	2022619	572	8900	46.6	2982	384.9x	74.8	9.9	WWAX00000000	SAMN13684483
Candidatus Aenigmarchaeota archaeon CS2-C132	Candidatus Aenigmarchaeia archaeon CS2-C132	1182387	204	35980	45.2	1553	658.4x	78.4	2.7	WVYL00000000	SAMN13684419
Candidatus Aenigmarchaeota archaeon A-C150	Candidatus Aenigmarchaeia archaeon A-C150	1053052	120	43266	46.2	1286	739.3x	78.3	8.4	WVWS00000000	SAMN13684374
Candidatus Aenigmarchaeota archaeon CS3-C152	Candidatus Aenigmarchaeia archaeon CS3-C152	774923	47	53107	46.0	903	1004.7x	73.7	0.9	WVZE00000000	SAMN13684438
Candidatus Bathyarchaeota archaeon CS4-C051	Candidatus Bathyarchaeia archaeon CS4-C051	2259871	578	2745	43.312	3367	344.5x	72.14	6.05	WVZV00000000	SAMN13684455
**GOK**											
Candidatus Bathyarchaeota archaeon CS4-K002	Candidatus Bathyarchaeia archaeon CS4-K002	1252559	395	4142	45.6	1882	372.8x	71.1	19.2	JAABYP000000000	SAMN13684274
Candidatus Bathyarchaeota archaeon KS3-K004	Candidatus Bathyarchaeia archaeon KS3-K004	1902980	383	10151	45.5	2541	245.5x	81.0	18.3	JAACAL000000000	SAMN13684322
Nitrosopumilaceae archaeon KS3-K007	Nitrosopumilaceae archaeon KS3-K007	1478333	214	39237	35.7	2150	316.2x	63.1	5.8	JAACAN000000000	SAMN13684324
Candidatus Bathyarchaeota archaeon CS1-K009	Candidatus Bathyarchaeia archaeon CS1-K009	820945	214	5893	48.3	1217	569.5x	56.7	5.6	JAABWV000000000	SAMN13684228
Candidatus Bathyarchaeota archaeon KS3-K019	Candidatus Bathyarchaeia archaeon KS3-K019	921282	240	5601	48.2	1393	507.4x	62.7	0.0	JAACAQ000000000	SAMN13684327
Thermoplasmata archaeon KS2-K019	Thermoplasmata archaeon KS2-K019	2971209	1204	2915	60.8	4850	157.3x	72.3	7.1	JAABZV000000000	SAMN13684306
Candidatus Korarchaeota archaeon KS3-K024	Candidatus Korarchaeia archaeon KS3-K024	3563601	898	9796	41.3	5504	131.2x	79.4	22.5	JAACAS000000000	SAMN13684329
Candidatus Bathyarchaeota archaeon CS3-K036	Candidatus Bathyarchaeia archaeon CS3-K036	1629506	571	3553	45.4	5270	286.9x	73.7	6.1	JAABXZ000000000	SAMN13684258
Thermoplasmata archaeon CS1-K061	Thermoplasmata archaeon CS1-K061	2747704	1098	3055	61.0	4472	170.1x	69.5	0.9	JAABWU000000000	SAMN13684227
Thermoplasmata archaeon CS4-K063	Thermoplasmata archaeon CS4-K063	2710710	1080	3062	60.9	4415	172.5x	68.4	0.9	JAABYY000000000	SAMN13684283
Nitrosopumilaceae archaeon CS3-K071	Nitrosopumilaceae archaeon CS3-K071	2658518	793	9479	34.5	4415	175.9x	64.2	9.2	JAABYG000000000	SAMN13684265
Thermoplasmata archaeon CS2-K079	Thermoplasmata archaeon CS2-K079	2726105	1107	2987	60.8	4487	171.5x	65.1	2.0	JAABXP000000000	SAMN13684248
Thermoplasmata archaeon KS1-K079	Thermoplasmata archaeon KS1-K079	2820546	1129	3007	60.9	4626	165.7x	69.8	1.7	JAABZN000000000	SAMN13684298
Thermoplasmata archaeon A-K083	Thermoplasmata archaeon A-K083	2814779	1139	2956	60.8	4620	166.1x	69.9	4.5	JAABWN000000000	SAMN13684220
Nitrosopumilaceae archaeon CS2-K084	Nitrosopumilaceae archaeon CS2-K084	2074561	577	22312	34.8	3455	225.3x	66.1	6.3	JAABXQ000000000	SAMN13684249
Thermoplasmata archaeon ACK-K091	Thermoplasmata archaeon ACK-K091	3464318	1331	3078	60.7	5610	134.9x	69.9	4.0	JAABVL000000000	SAMN13684192
Thermoplasmata archaeon KS4-K091	Thermoplasmata archaeon KS4-K091	3464318	1331	3078	60.7	5610	134.9x	69.9	4.5	JAACBQ000000000	SAMN13684353
Candidatus Bathyarchaeota archaeon CS3-K092	Candidatus Bathyarchaeia archaeon CS3-K092	1020515	311	4993	47.7	1621	458.1x	63.6	0.5	JAABYN000000000	SAMN13684272
Candidatus Bathyarchaeota archaeon CS2-K094	Candidatus Bathyarchaeia archaeon CS2-K094	1026952	321	4623	47.7	1635	455.2x	57.5	0.0	JAABXT000000000	SAMN13684252
Thermoplasmata archaeon KS3-K094	Thermoplasmata archaeon KS3-K094	2844697	1140	3001	60.8	4664	164.3x	69.0	4.1	JAACAZ000000000	SAMN13684336
Candidatus Bathyarchaeota archaeon A-K098	Candidatus Bathyarchaeia archaeon A-K098	851142	247	5039	47.5	1378	549.3x	53.3	0.0	JAABWS000000000	SAMN13684225
Nitrosopumilaceae archaeon ACK-K098	Nitrosopumilaceae archaeon ACK-K098	2579314	809	8280	34.8	4410	181.3x	65.1	7.8	JAABWC000000000	SAMN13684209
Nitrosopumilaceae archaeon KS4-K098	Nitrosopumilaceae archaeon KS4-K098	2579314	809	8280	34.8	4410	181.3x	65.1	7.8	JAACBS000000000	SAMN13684355
Candidatus Korarchaeota archaeon A-K099	Candidatus Korarchaeia archaeon A-K099	2808495	587	13147	41.4	4150	166.5x	75.9	14.7	JAABWT000000000	SAMN13684226

**Online-only Table 2:** Genomic properties of the 270 bacterial MAGs reported in the study.

Bin taxonomy (NCBI)	GTDB taxonomy	Size (bp)	No. of contigs	N50	GC (%)	No. of PEGs (RAST)	Genome coverage	Completeness (%)	Contamination (%)	Genome accession (NCBI)	Biosample accession
**GOC**											
Gammaproteobacteria bacterium CS3-C001	Gammaproteobacteria bacterium CS3-C001	2168098	90	198546	48.62	2302	359.1x	96.33	1.33	WVYN00000000	SAMN13684421
Armatimonadetes bacterium CS1-C002	Armatimonadota bacterium CS1-C002	2679535	253	29050	55.936	3079	290.5x	84.72	0.93	WVXJ00000000	SAMN13684391
Gammaproteobacteria bacterium CS3-C002	Gammaproteobacteria bacterium CS3-C002	3848882	86	74003	64.593	3933	202.3x	88.64	4.53	WVYO00000000	SAMN13684422
Deltaproteobacteria bacterium CS1-C003	Deltaproteobacteria bacterium CS1-C003	2496075	115	81482	54.895	2696	311.9x	92.02	1.68	WVXK00000000	SAMN13684392
Gammaproteobacteria bacterium ACK-C003	Gammaproteobacteria bacterium ACK-C003	3395771	183	98032	64.133	3576	229.3x	90.94	6.76	WVWT00000000	SAMN13684375
Gammaproteobacteria bacterium KS4-C003	Gammaproteobacteria bacterium KS4-C003	3395771	183	74003	64.133	3576	229.3x	86.21	6.9	WWBK00000000	SAMN13684496
Proteobacteria bacterium CS4-C004	Proteobacteria bacterium CS4-C004	5718666	751	16461	50.267	7730	136.1x	85.81	9.74	WVZF00000000	SAMN13684439
Gemmatimonadetes bacterium KS2-C005	Gemmatimonadetes bacterium KS2-C005	3586048	199	62373	64.602	3422	217.1x	77.61	7.69	WWAP00000000	SAMN13684475
Gammaproteobacteria bacterium ACK-C006	Gammaproteobacteria bacterium ACK-C006	2340594	177	360840	48.125	2570	332.4x	74.75	3.16	WVWU00000000	SAMN13684376
Gammaproteobacteria bacterium KS4-C006	Gammaproteobacteria bacterium KS4-C006	2340594	177	102188	48.125	2570	332.4x	100	4.67	WWBL00000000	SAMN13684497
Proteobacteria bacterium CS4-C006	Proteobacteria bacterium CS4-C006	4614671	452	22180	50.159	5892	168.7x	84.15	9.52	WVZG00000000	SAMN13684440
Woeseiaceae bacterium CS3-C006	Woeseiaceae bacterium CS3-C006	3264774	46	121008	59.637	3154	238.5x	82.83	2.14	WVYQ00000000	SAMN13684424
Anaerolineales bacterium CS4-C007	Anaerolineales bacterium CS4-C007	5573343	589	45216	53.098	6050	139.7x	87.27	5.45	WVZH00000000	SAMN13684441
Candidatus Dadabacteria bacterium CS3-C007	Candidatus Dadabacteria bacterium CS3-C007	1738464	689	16166	39.714	2541	447.8x	88.03	13	WVYR00000000	SAMN13684425
Hydrogenophaga sp. KS3-C007	Hydrogenophaga sp. KS3-C007	4792363	66	171063	69.119	4601	162.5x	96.06	9.4	WWBB00000000	SAMN13684487
Anaerolineae bacterium CS1-C008	Anaerolineae bacterium CS1-C008	4740090	1440	4770	57.832	6101	164.2x	70.61	8.8	WVXL00000000	SAMN13684393
Xanthomonadales bacterium ACK-C008	Xanthomonadales bacterium ACK-C008	2725236	165	43476	66.456	2738	285.7x	89.13	3.74	WVWV00000000	SAMN13684377
Xanthomonadales bacterium KS4-C008	Xanthomonadales bacterium KS4-C008	2725236	165	43476	66.456	2738	285.7x	89.13	3.74	WWBM00000000	SAMN13684498
Candidatus Latescibacteria bacterium CS1-C010	Krumholzibacteria bacterium CS1-010	4149188	81	131317	52.814	3712	187.6x	88.95	2.2	WVXM00000000	SAMN13684394
Deltaproteobacteria bacterium CS3-C011	Deltaproteobacteria bacterium CS3-C011	2705148	179	60087	54.801	2903	287.8x	92.02	0.84	WVYS00000000	SAMN13684426
Gammaproteobacteria bacterium A-C011	Gammaproteobacteria bacterium A-C011	3817258	120	74003	64.482	3939	203.9x	88.84	5.53	WVWI00000000	SAMN13684364
Gammaproteobacteria bacterium KS2-C012	Gammaproteobacteria bacterium KS2-C012	3800388	107	74003	64.518	3927	204.9x	89.23	5.62	WWAQ00000000	SAMN13684476
Acidobacteria bacterium ACK-C013	Acidobacteria bacterium ACK-C013	3311811	321	22375	65.755	3285	235.1x	82.48	5.78	WVWW00000000	SAMN13684378
Acidobacteria bacterium KS4-C013	Acidobacteria bacterium KS4-C013	3311811	321	22375	65.755	3285	235.1x	82.48	5.78	WWBN00000000	SAMN13684499
Gammaproteobacteria bacterium KS1-C013	Gammaproteobacteria bacterium KS1-C013	3876872	127	74003	64.449	3988	200.8x	88.84	6.38	WWAB00000000	SAMN13684461
Pseudomonas-A stutzeri CS4-C014	Pseudomonas-A stutzeri CS4-C014	4672359	76	665743	64.115	4490	166.6x	96.55	3.45	WVZJ00000000	SAMN13684443
Candidatus Aminicenantes bacterium CS1-C017	Aminicenantaceae bacterium CS1-C017	2591933	74	73629	41.443	2585	300.4x	82.25	2.56	WVXO00000000	SAMN13684396
Candidatus Aminicenantes bacterium CS4-C017	Aminicenantaceae bacterium CS4-C017	3142456	204	42592	42.142	3153	247.7x	89.52	6.2	WVZK00000000	SAMN13684444
Candidatus Aminicenantes bacterium CS4-C018	Aminicenantaceae bacterium CS4-C018	3276286	49	135889	44.281	3071	237.6x	86.1	4.26	WVZL00000000	SAMN13684445
Xanthomonadales bacterium CS3-C018	Xanthomonadales bacterium CS3-C018	2719212	165	43476	66.447	2727	286.3x	88.77	4.29	WVYT00000000	SAMN13684427
Woeseiaceae bacterium ACK-C019	Woeseiaceae bacterium ACK-C019	2777859	159	227966	59.062	2893	280.3x	74.03	3.19	WVWX00000000	SAMN13684379
Woeseiaceae bacterium KS4-C019	Woeseiaceae bacterium KS4-C019	2777859	159	227966	59.062	2893	280.3x	74.03	3.19	WWBO00000000	SAMN13684500
Anaerolineales bacterium CS4-C020	Anaerolineales bacterium CS4-C020	5236009	152	94085	49.614	5100	148.7x	82.73	7.45	WVZM00000000	SAMN13684446
Gammaproteobacteria bacterium KS3-C020	Gammaproteobacteria bacterium KS3-C020	3380137	111	73841	64.558	3511	230.3x	83.89	3.94	WWBC00000000	SAMN13684488
Acidobacteria bacterium KS1-C022	Acidobacteria bacterium KS1-C022	3275514	400	20795	65.516	3358	237.7x	76.2	9.57	WWAC00000000	SAMN13684462
Gammaproteobacteria bacterium CS2-C022	Gammaproteobacteria bacterium CS2-C022	3986235	203	73841	64.236	4225	195.3x	88.68	5.88	WVYE00000000	SAMN13684412
Hydrogenophaga sp. CS4-C022	Hydrogenophaga sp. CS4-C022	4654145	51	171063	69.144	4469	167.3x	96.5	0.47	WVZN00000000	SAMN13684447
Xanthomonadales bacterium A-C023	Xanthomonadales bacterium A-C023	2566916	153	43476	66.533	2579	303.3x	84.33	6.9	WVWJ00000000	SAMN13684365
Candidatus Aminicenantes bacterium CS4-C025	Aminicenantia bacterium CS4-C025	10383395	768	26679	43.532	10075	75x	90.47	7.55	WVZO00000000	SAMN13684448
Gammaproteobacteria bacterium A-C027	Gammaproteobacteria bacterium A-C027	2519335	294	102188	47.563	2987	309x	96.8	3.98	WVWK00000000	SAMN13684366
Proteobacteria bacterium CS1-C027	Proteobacteria bacterium CS1-C027	3725068	292	20857	50.713	4760	209x	72.65	3.87	WVXP00000000	SAMN13684397
Armatimonadetes bacterium CS4-C029	Armatimonadota bacterium CS4-C029	2040739	167	31676	55.771	2312	381.5x	77.78	1.54	WVZP00000000	SAMN13684449
Gemmatimonadetes bacterium CS1-C030	Gemmatimonadetes bacterium CS1-C030	3622874	131	62636	64.681	3380	214.9x	76.86	5.49	WVXQ00000000	SAMN13684398
Gemmatimonadales bacterium CS4-C031	Gemmatimonadales bacterium CS4-C031	5768264	723	32750	63.574	5992	135x	73.08	8.34	WVZQ00000000	SAMN13684450
Candidatus Dadabacteria bacterium ACK-C033	Candidatus Dadabacteria bacterium ACK-C033	2803315	626	6723	33.636	3831	277.7x	91.11	8.1	WVWZ00000000	SAMN13684381
Acidobacteria bacterium CS2-C035	Acidobacteria bacterium CS2-C035	3177451	424	21277	65.221	3303	245x	73.99	5.98	WVYG00000000	SAMN13684414
Xanthomonadales bacterium KS1-C035	Xanthomonadales bacterium KS1-C035	2716558	179	43476	66.314	2739	286.6x	88.58	4.41	WWAD00000000	SAMN13684463
Candidate division Zixibacteria bacterium CS4-C036	Candidate division Zixibacteria bacterium CS4-C036	5539247	1197	9706	49.152	7424	140.5x	88.7	7.41	WVZS00000000	SAMN13684452
Pseudomonas-A stutzeri A-C038	Pseudomonas-A stutzeri A-C038	4499457	34	665743	64.126	4297	173x	96.17	0.91	WVWL00000000	SAMN13684367
Candidatus Dadabacteria bacterium CS3-C039	Candidatus Dadabacteria bacterium CS3-C039	3395967	962	153898	32.816	5340	229.2x	97.74	2.2	WVYU00000000	SAMN13684428
Hydrogenophaga sp. A-C039	Hydrogenophaga sp. A-C039	4716175	84	171063	69.066	4563	165.1x	96.03	1.52	WVWM00000000	SAMN13684368
Hydrotalea flava CS4-C041	Hydrotalea flava CS4-C041	3190995	301	16933	36.868	3568	244x	92.59	0	WVZT00000000	SAMN13684453
Gammaproteobacteria bacterium KS3-C044	Gammaproteobacteria bacterium KS3-C044	3102346	755	52159	46.477	4339	250.9x	84.24	8.6	WWBD00000000	SAMN13684489
Xanthomonadales bacterium KS2-C044	Xanthomonadales bacterium KS2-C044	2757556	250	43476	66.221	2844	282.3x	86.59	5.35	WWAR00000000	SAMN13684477
Planctomycetales bacterium CS4-C045	Pirellulales bacterium CS4-C045	3327317	437	11342	61.033	3638	234x	90.8	3.79	WVZU00000000	SAMN13684454
Pseudomonas-A stutzeri ACK-C047	Pseudomonas-A stutzeri ACK-C047	4480624	52	665743	64.071	4319	173.8x	96.17	1.25	WVXA00000000	SAMN13684382
Pseudomonas-A stutzeri KS4-C047	Pseudomonas-A stutzeri KS4-C047	4480624	52	665743	64.071	4319	173.8x	96.17	1.25	WWBQ00000000	SAMN13684502
Hydrogenophaga sp. ACK-C049	Hydrogenophaga sp. ACK-C049	4688826	83	171063	69.057	4526	166x	96.03	0.93	WVXB00000000	SAMN13684383
Hydrogenophaga sp. KS4-C049	Hydrogenophaga sp. KS4-C049	4688826	83	171063	69.057	4526	166x	96.03	0.93	WWBR00000000	SAMN13684503
Xanthomonadales bacterium CS2-C049	Xanthomonadales bacterium CS2-C049	3162922	404	36822	65.467	3538	246.1x	88.41	7.74	WVYH00000000	SAMN13684415
Anaerolineae bacterium KS2-C051	Anaerolineae bacterium KS2-C051	6346080	1552	16118	56.98	8617	122.7x	85.63	9.58	WWAS00000000	SAMN13684478
Gemmatimonadales bacterium KS3-C054	Gemmatimonadales bacterium KS3-C054	4194012	298	53486	64.231	4133	185.6x	84.09	7.21	WWBE00000000	SAMN13684490
Gemmatimonadales bacterium ACK-C060	Gemmatimonadales bacterium ACK-C060	3409282	223	63862	64.348	3331	228.4x	75.78	7.21	WVXC00000000	SAMN13684384
Gemmatimonadales bacterium KS4-C060	Gemmatimonadales bacterium KS4-C060	3409282	223	63862	64.348	3331	228.4x	75.78	7.21	WWBS00000000	SAMN13684504
Planctomycetales bacterium CS3-C060	Pirellulales bacterium CS3-C060	3417275	1051	4525	63.049	4321	227.8x	70.57	5.38	WVYV00000000	SAMN13684429
Gammaproteobacteria bacterium KS1-C062	Gammaproteobacteria bacterium KS1-C062	2727925	441	89163	47.235	3429	285.4x	96.38	6.22	WWAE00000000	SAMN13684464
Planctomycetales bacterium CS3-C063	Pirellulales bacterium CS3-C063	4259264	968	8656	60.173	5237	182.8x	91.95	9.89	WVYW00000000	SAMN13684430
Gemmatimonadales bacterium CS3-C064	Gemmatimonadales bacterium CS3-C064	4239130	351	51911	64.143	4196	183.7x	84.39	3.3	WVYX00000000	SAMN13684431
Planctomycetales bacterium ACK-C064	Pirellulales bacterium ACK-C064	3262357	481	10931	60.818	3638	238.6x	85.92	2.3	WVXD00000000	SAMN13684385
Planctomycetales bacterium KS4-C064	Pirellulales bacterium KS4-C064	3262357	481	10931	60.818	3638	238.6x	85.92	2.3	WWBT00000000	SAMN13684505
Planctomycetales bacterium CS1-C065	Pirellulales bacterium CS1-C065	3194297	427	11705	61.099	3482	243.7x	87.36	4.94	WVXS00000000	SAMN13684400
Hydrogenophaga sp. CS3-C067	Hydrogenophaga sp. CS3-C067	4691942	83	171063	69.053	4533	165.9x	96.03	0.93	WVYY00000000	SAMN13684432
Candidatus bacterium CS1-C068	Candidatus bacterium CS1-C068	1325588	101	14971	42.628	1452	587.3x	65.1	10.78	WVXT00000000	SAMN13684401
Gemmatimonadales bacterium CS1-C069	Gemmatimonadales bacterium CS1-C069	5243692	788	41299	63.318	5770	148.5x	84.25	7.37	WVXU00000000	SAMN13684402
Pseudomonas-A stutzeri CS3-C070	Pseudomonas-A stutzeri CS3-C070	4492264	63	665743	64.043	4304	173.3x	96.17	1.06	WVYZ00000000	SAMN13684433
Gammaproteobacteria bacterium CS1-C072	Gammaproteobacteria bacterium CS1-C072	3840661	95	74003	64.532	3944	202.7x	88.64	4.88	WVXV00000000	SAMN13684403
Hydrogenophaga sp. CS2-C072	Hydrogenophaga sp. CS2-C072	4723047	91	171063	69.055	4567	164.8x	96.03	1.52	WVYI00000000	SAMN13684416
Planctomycetales bacterium KS3-C073	Pirellulales bacterium KS3-C073	4203496	937	8852	60.252	5126	185.2x	94.25	7.11	WWBF00000000	SAMN13684491
Deltaproteobacteria bacterium CS3-C074	Deltaproteobacteria bacterium CS3-C074	2209352	357	15977	51.268	2744	352.4x	79.41	9.01	WVZA00000000	SAMN13684434
Candidatus bacterium KS1-C076	Candidatus bacterium KS1-C076	1954622	324	9368	42.72	2304	398.3x	91.11	19.4	WWAF00000000	SAMN13684465
Hydrogenophaga sp. KS2-C076	Hydrogenophaga sp. KS2-C076	4706689	84	171063	69.058	2304	165.4x	96.03	1.52	WWAT00000000	SAMN13684479
Pseudomonas-A stutzeri CS2-C076	Pseudomonas-A stutzeri CS2-C076	4514078	78	665743	64.02	4372	172.5x	96.17	0.85	WVYJ00000000	SAMN13684417
Candidate division Zixibacteria bacterium A-C077	Candidate division Zixibacteria bacterium A-C077	2679588	91	91123	62.223	2814	290.5x	96.52	1.1	WVWN00000000	SAMN13684369
Planctomycetales bacterium KS1-C079	Pirellulales bacterium KS1-C079	3323674	486	10931	60.874	3701	234.2x	86.21	6.87	WWAG00000000	SAMN13684466
Candidatus Latescibacteria bacterium KS2-C080	Krumholzibacteria bacterium KS2-C080	2728096	79	13031	53.021	2480	285.4x	78.79	6.44	WWAU00000000	SAMN13684480
Hydrogenophaga sp. KS1-C080	Hydrogenophaga sp. KS1-C080	4728702	98	171063	69.032	4578	164.6x	96.03	1.75	WWAH00000000	SAMN13684467
Anaerolineae bacterium KS3-C083	Anaerolineae bacterium KS3-C083	6425225	1585	15113	57.108	8715	121.2x	82.87	7.88	WWBG00000000	SAMN13684492
Gammaproteobacteria bacterium CS4-C084	Gammaproteobacteria bacterium CS4-C084	3803886	99	74003	64.513	3903	204.7x	88.64	5	WVZX00000000	SAMN13684457
Pseudomonas-A stutzeri KS1-C084	Pseudomonas-A stutzeri KS1-C084	4486979	61	665743	64.062	4332	173.5x	96.17	0.38	WWAI00000000	SAMN13684468
Pseudomonas-A stutzeri KS2-C084	Pseudomonas-A stutzeri KS2-C084	4442226	24	665743	64.151	4234	175.3x	96.17	0.14	WWAV00000000	SAMN13684481
Candidatus Latescibacteria bacterium A-C086	Krumholzibacteria bacterium A-C086	3153095	74	131317	52.876	2842	246.9x	97.74	2.2	WVWO00000000	SAMN13684370
Candidatus Latescibacteria bacterium KS3-C087	Krumholzibacteria bacterium KS3-C087	2702945	84	3919	52.961	2459	288x	72.07	13.87	WWBH00000000	SAMN13684493
Proteobacteria bacterium CS1-C090	Proteobacteria bacterium CS1-C090	3777191	233	55222	51.237	4369	206.1x	77.29	7.26	WVXX00000000	SAMN13684405
Planctomycetales bacterium KS2-C091	Pirellulales bacterium KS2-C091	3301600	513	10931	60.657	3701	235.8x	87.07	5.38	WWAW00000000	SAMN13684482
Candidatus Latescibacteria bacterium CS3-C092	Krumholzibacteria bacterium CS3-C092	3413421	142	131317	52.677	3188	228.1x	91.14	2.2	WVZB00000000	SAMN13684435
Candidatus Latescibacteria bacterium ACK-C097	Krumholzibacteria bacterium ACK-C097	2185789	207	153898	52.507	2251	356.2x	97.74	4.4	WVXE00000000	SAMN13684386
Candidatus Latescibacteria bacterium KS4-C097	Krumholzibacteria bacterium KS4-C097	2185789	207	5514	52.507	2251	356.2x	53.62	1.69	WWBU00000000	SAMN13684506
Candidatus Aminicenantes bacterium CS1-C098	Aminicenantia bacterium CS1-C098	10782236	930	25864	43.342	10796	72.2x	90.47	8.55	WVXY00000000	SAMN13684406
Armatimonadetes bacterium A-C102	Armatimonadota bacterium A-C102	2287921	300	28335	55.573	2735	340.3x	77.78	7.2	WVWP00000000	SAMN13684371
Deltaproteobacteria bacterium KS1-C103	Deltaproteobacteria bacterium KS1-C103	5105547	1494	5224	52.216	7450	152.5x	76.57	8.67	WWAJ00000000	SAMN13684469
Anaerolineae bacterium CS2-C105	Anaerolineae bacterium CS2-C105	6094619	1425	16962	57.2	8094	127.7x	86.54	9.07	WVYK00000000	SAMN13684418
Candidatus Latescibacteria bacterium CS4-C107	Krumholzibacteria bacterium CS4-C107	4128822	81	121452	52.749	3721	188.6x	75.21	1.1	WVZY00000000	SAMN13684458
Acidobacteria bacterium CS1-C108	Acidobacteria bacterium CS1-C108	4034700	657	19042	64.88	4355	193x	86.8	7.75	WVXZ00000000	SAMN13684407
Hydrotalea flava A-C108	Hydrotalea flava A-C108	3169773	301	16667	36.887	3545	245.6x	92.59	0.99	WVWQ00000000	SAMN13684372
Candidatus Saccharibacteria bacterium CS3-C112	Patescibacteria bacterium CS3-C112	1074178	294	33328	48.205	1644	724.8x	83.62	5.45	WVZC00000000	SAMN13684436
Burkholderiales bacterium KS1-C116	Burkholderiales bacterium KS1-C116	4461420	1674	3544	52.795	7847	174.5x	72.18	6.3	WWAK00000000	SAMN13684470
Hydrogenophaga sp. CS1-C118	Hydrogenophaga sp. CS1-C118	4666132	73	171063	69.11	4483	166.8x	95.56	1.52	WVYA00000000	SAMN13684408
Armatimonadetes bacterium ACK-C119	Armatimonadota bacterium ACK-C119	3238322	689	20765	54.923	4194	240.4x	87.04	8.75	WVXF00000000	SAMN13684387
Armatimonadetes bacterium KS3-C119	Armatimonadota bacterium KS3-C119	2192828	249	30302	55.668	2563	355x	77.78	3.5	WWBI00000000	SAMN13684494
Armatimonadetes bacterium KS4-C119	Armatimonadota bacterium KS4-C119	3238322	689	20765	54.923	4194	240.4x	87.04	8.75	WWBV00000000	SAMN13684507
Candidatus Aminicenantes bacterium A-C121	Aminicenantia bacterium A-C121	11373454	1143	25427	43.407	11625	68.5x	93.03	8.76	WVWR00000000	SAMN13684373
Candidatus Latescibacteria bacterium KS1-C122	Krumholzibacteria bacterium KS1-C122	3116952	86	2382	52.806	2845	249.8x	78.65	26.94	WWAL00000000	SAMN13684471
Armatimonadetes bacterium KS2-C125	Armatimonadota bacterium KS2-C125	2240368	266	29718	55.623	2646	347.5x	77.78	7.2	WWAY00000000	SAMN13684484
Acidobacteria bacterium CS4-C126	Acidobacteria bacterium CS4-C126	3974284	674	18405	64.8	4493	194.7x	82.95	9.46	WVZZ00000000	SAMN13684459
Candidatus bacterium ACK-C128	Candidatus bacterium ACK-C128	1429657	148	8589	42.59	1631	544.6x	76.46	8.72	WVXG00000000	SAMN13684388
Candidatus bacterium KS4-C128	Candidatus bacterium KS4-C128	1429657	148	16166	42.59	1631	544.6x	88.03	13	WWBW00000000	SAMN13684508
Pseudomonas-A stutzeri CS1-C129	Pseudomonas-A stutzeri CS1-C129	4509433	76	665743	64.007	4332	172.6x	96.17	0.66	WVYB00000000	SAMN13684409
Hydrotalea flava ACK-C130	Hydrotalea flava ACK-C130	3193437	316	16502	36.848	3584	243.8x	92.76	0.66	WVXH00000000	SAMN13684389
Hydrotalea flava KS4-C130	Hydrotalea flava KS4-C130	3193437	316	16502	36.848	3584	243.8x	92.76	0.66	WWBX00000000	SAMN13684509
Candidatus Aminicenantes bacterium ACK-C131	Aminicenantia bacterium ACK-C131	11488117	1172	25427	43.414	11735	67.8x	93.03	8.81	WVXI00000000	SAMN13684390
Candidatus Aminicenantes bacterium KS2-C131	Aminicenantia bacterium KS2-C131	10513167	839	26137	43.47	10337	74.1x	90.47	7.91	WWAZ00000000	SAMN13684485
Candidatus Aminicenantes bacterium KS4-C131	Aminicenantia bacterium KS4-C131	10499685	838	26137	43.484	10334	74.1x	90.47	7.53	WWBY00000000	SAMN13684510
Xanthomonadales bacterium CS1-C135	Xanthomonadales bacterium CS1-C135	2712242	188	43476	66.198	2753	287x	87.5	4.47	WVYC00000000	SAMN13684410
Candidatus Aminicenantes bacterium KS3-C136	Aminicenantia bacterium KS3-C136	10419537	807	26536	43.507	10177	74.7x	90.47	7.91	WWBJ00000000	SAMN13684495
Hydrotalea flava CS3-C139	Hydrotalea flava CS3-C139	3179459	308	16667	36.871	3563	244.9x	92.59	0.99	WVZD00000000	SAMN13684437
Hydrotalea flava KS2-C139	Hydrotalea flava KS2-C139	3163410	298	16667	36.885	3539	246.1x	92.59	0	WWBA00000000	SAMN13684486
Xanthomonadales bacterium CS4-C139	Xanthomonadales bacterium CS4-C139	3001128	376	41092	65.678	3208	259.4x	88.32	6.2	WWAA00000000	SAMN13684460
Hydrotalea flava CS1-C145	Hydrotalea flava CS1-C145	3185564	313	16667	36.845	3581	244.4x	92.59	0	WVYD00000000	SAMN13684411
Candidatus Aminicenantes bacterium CS2-C146	Aminicenantia bacterium CS2-C146	11264024	1088	25482	43.458	11401	69.1x	93.03	8.39	WVYM00000000	SAMN13684420
Armatimonadetes bacterium KS1-C150	Armatimonadota bacterium KS1-C150	2248654	273	29718	55.65	2638	346.2x	77.93	5.82	WWAM00000000	SAMN13684472
Hydrotalea flava KS1-C152	Hydrotalea flava KS1-C152	3201758	307	16933	36.85	3597	243.2x	92.59	0.49	WWAN00000000	SAMN13684473
Candidatus Aminicenantes bacterium KS1-C158	Aminicenantia bacterium KS1-C158	10495492	832	26137	43.486	10314	74.2x	90.47	7.48	WWAO00000000	SAMN13684474
**GOK**											
Deltaproteobacteria bacterium KS2-K001	Deltaproteobacteria bacterium KS2-K001	5194107	1099	9819	49.218	7306	89.9x	84.02	4.75	JAABZR000000000	SAMN13684302
Desulfuromonadales bacterium KS3-K001	Desulfuromonadales bacterium KS3-K001	1624735	190	15014	60.799	1970	287.7x	52.63	0	JAACAJ000000000	SAMN13684320
Gammaproteobacteria bacterium CS3-K001	Gammaproteobacteria bacterium CS3-K001	2179105	98	360840	48.799	2313	214.5x	96.98	1.08	JAABXV000000000	SAMN13684254
Gammaproteobacteria bacterium CS3-K002	Gammaproteobacteria bacterium CS3-K002	5214606	716	86349	64.087	5636	89.6x	100	8.73	JAABXW000000000	SAMN13684255
Gemmatimonadetes bacterium KS3-K002	Gemmatimonadetes bacterium KS3-K002	3760514	149	91869	66.586	3549	124.3x	91.54	4.4	JAACAK000000000	SAMN13684321
Phycisphaerae bacterium KS2-K004	Phycisphaerae bacterium KS2-K004	6730037	744	22861	47.305	6705	69.5x	92.61	10.65	JAABZS000000000	SAMN13684303
Candidate division KSB1 bacterium KS3-K005	Candidate division KSB1 bacterium KS3-K005	5819846	507	21511	47.171	6424	80.3x	89.98	16.35	JAACAM000000000	SAMN13684323
Gammaproteobacteria bacterium ACK-K006	Gammaproteobacteria bacterium ACK-K006	3947980	74	30736	64.681	3938	118.4x	52.38	2.05	JAABVM000000000	SAMN13684193
Gammaproteobacteria bacterium KS2-K006	Gammaproteobacteria bacterium KS2-K006	4510068	201	65644	66.284	4430	103.7x	82.64	13.56	JAABZT000000000	SAMN13684304
Gammaproteobacteria bacterium KS4-K006	Gammaproteobacteria bacterium KS4-K006	3947980	74	98032	64.681	3938	118.4x	91.12	6.4	JAACBB000000000	SAMN13684338
Gammaproteobacteria bacterium KS3-K009	Gammaproteobacteria bacterium KS3-K009	2746408	592	19975	65.325	3287	170.2x	73.51	11.77	JAACAO000000000	SAMN13684325
Gemmatimonadetes bacterium CS2-K009	Gemmatimonadetes bacterium CS2-K009	3544438	850	57180	65.381	4247	131.9x	74.1	17.61	JAABXI000000000	SAMN13684241
Woeseiaceae bacterium CS3-K009	Woeseiaceae bacterium CS3-K009	2288386	183	118102	59.105	2391	204.3x	53.89	3.03	JAABXX000000000	SAMN13684256
Gammaproteobacteria bacterium CS2-K010	Gammaproteobacteria bacterium CS2-K010	3281353	671	92672	47.864	3893	142.5x	98.28	12.65	JAABXJ000000000	SAMN13684242
Gemmatimonadetes bacterium KS3-K010	Gemmatimonadetes bacterium KS3-K010	4205843	1191	4609	70.209	4992	111.2x	82.32	6.59	JAACAP000000000	SAMN13684326
Candidate division Zixibacteria bacterium KS2-K011	Candidate division Zixibacteria bacterium KS2-K011	3663752	655	10946	46.205	4262	127.6x	90.59	2.88	JAABZU000000000	SAMN13684305
Gammaproteobacteria bacterium KS1-K011	Gammaproteobacteria bacterium KS1-K011	4014915	106	98032	64.646	4039	116.4x	90.41	6.58	JAABZC000000000	SAMN13684287
Pseudomonas-A stutzeri CS4-K011	Pseudomonas-A stutzeri CS4-K011	3943168	1645	2689	61.88	6400	118.6x	57.71	6.13	JAABYQ000000000	SAMN13684275
Gammaproteobacteria bacterium ACK-K012	Gammaproteobacteria bacterium ACK-K012	1835019	178	73626	48.317	2229	254.8x	56.37	5.08	JAABVN000000000	SAMN13684194
Gammaproteobacteria bacterium CS2-K012	Gammaproteobacteria bacterium CS2-K012	4099482	103	98916	64.678	4106	114x	90.41	6.45	JAABXK000000000	SAMN13684243
Gammaproteobacteria bacterium KS4-K012	Gammaproteobacteria bacterium KS4-K012	1835019	178	360840	48.317	2229	254.8x	76.71	4.64	JAACBC000000000	SAMN13684339
Gemmatimonadetes bacterium A-K012	Gemmatimonadetes bacterium A-K012	5043435	1888	3532	68.646	7052	92.7x	61.16	10.5	JAABWD000000000	SAMN13684210
Phycisphaerae bacterium CS1-K013	Phycisphaerae bacterium CS1-K013	6462991	911	17406	47.025	6709	72.3x	96.49	40.88	JAABWW000000000	SAMN13684229
Xanthomonadales bacterium ACK-K013	Xanthomonadales bacterium ACK-K013	2736368	700	6814	62.971	3357	170.8x	84.33	31.82	JAABVO000000000	SAMN13684195
Xanthomonadales bacterium KS4-K013	Xanthomonadales bacterium KS4-K013	2736368	700	6814	62.971	3357	170.8x	84.33	31.82	JAACBD000000000	SAMN13684340
Pseudomonadales bacterium ACK-K015	Pseudomonadales bacterium ACK-K015	3544185	493	41843	64.303	3852	131.9x	64.16	6.47	JAABVP000000000	SAMN13684196
Pseudomonadales bacterium KS4-K015	Pseudomonadales bacterium KS4-K015	3544185	493	41843	64.303	3852	131.9x	64.16	6.47	JAACBE000000000	SAMN13684341
Gammaproteobacteria bacterium CS1-K016	Gammaproteobacteria bacterium CS1-K016	4155143	105	95449	64.513	4171	112.5x	91.12	6.13	JAABWX000000000	SAMN13684230
Phycisphaerae bacterium CS4-K016	Phycisphaerae bacterium CS4-K016	6782154	997	24008	46.969	7085	68.9x	65.34	7.62	JAABYR000000000	SAMN13684276
Actinobacteria bacterium CS3-K018	Microtrichales bacterium CS3-K018	3784163	1765	2347	66.876	5990	123.5x	71.04	16.62	JAABXY000000000	SAMN13684257
Candidatus Dadabacteria bacterium ACK-K020	Candidatus Dadabacteria bacterium ACK-K020	2238320	236	14971	39.583	2674	208.9x	70.56	14.01	JAABVQ000000000	SAMN13684197
Candidatus Dadabacteria bacterium KS4-K020	Candidatus Dadabacteria bacterium KS4-K020	2238320	236	153898	39.583	2674	208.9x	97.74	4.5	JAACBF000000000	SAMN13684342
Nitrospinaceae bacterium KS3-K021	Nitrospinaceae bacterium KS3-K021	4488294	1359	4443	54.059	6463	104.2x	89.66	34.42	JAACAR000000000	SAMN13684328
Actinobacteria bacterium CS2-K022	Microtrichales bacterium CS2-K022	3810679	1819	2224	66.554	6158	122.7x	67.02	11.25	JAABXL000000000	SAMN13684244
Actinobacteria bacterium KS1-K023	Microtrichales bacterium KS1-K023	3656183	1695	2349	66.98	5752	127.9x	68.4	10.71	JAABZD000000000	SAMN13684288
Gammaproteobacteria bacterium A-K023	Gammaproteobacteria bacterium A-K023	4056643	132	98032	64.569	4111	115.2x	91.12	6.4	JAABWE000000000	SAMN13684211
Gammaproteobacteria bacterium KS2-K023	Gammaproteobacteria bacterium KS2-K023	4088207	87	98032	64.552	4099	114.4x	90.41	6.48	JAABZW000000000	SAMN13684307
Gemmatimonadetes bacterium CS1-K023	Gemmatimonadetes bacterium CS1-K023	3856915	405	86694	65.716	3910	121.2x	91.14	15.81	JAABWY000000000	SAMN13684231
Gemmatimonadetes bacterium CS4-K023	Gemmatimonadetes bacterium CS4-K023	3411065	320	86694	65.924	3900	137.1x	58.62	6.03	JAABYS000000000	SAMN13684277
Gammaproteobacteria bacterium CS4-K024	Gammaproteobacteria bacterium CS4-K024	3084059	59	98916	65.043	3060	151.6x	81.03	3.45	JAABYT000000000	SAMN13684278
Gemmatimonadetes bacterium ACK-K026	Gemmatimonadetes bacterium ACK-K026	4675278	1776	3485	68.498	6631	100x	69.39	18.57	JAABVR000000000	SAMN13684198
Gemmatimonadetes bacterium KS4-K026	Gemmatimonadetes bacterium KS4-K026	4675278	1776	3532	68.498	6630	100x	61.16	10.5	JAACBG000000000	SAMN13684343
Gemmatimonadetes bacterium KS2-K027	Gemmatimonadetes bacterium KS2-K027	3980195	383	90624	65.856	4015	117.5x	91.14	12.2	JAABZX000000000	SAMN13684308
Actinobacteria bacterium ACK-K028	Microtichales bacterium ACK-K028	3683349	1713	16116	66.954	5834	126.9x	63.04	12.96	JAABVS000000000	SAMN13684199
Actinobacteria bacterium KS4-K028	Microtrichales bacterium KS4-K028	3683349	1713	16116	66.954	5834	126.9x	70.42	14.1	JAACBH000000000	SAMN13684344
Gammaproteobacteria bacterium A-K032	Gammaproteobacteria bacterium A-K032	1581429	65	360840	48.621	1772	295.6x	76.71	4.64	JAABWF000000000	SAMN13684212
Gemmatimonadetes bacterium KS2-K035	Gemmatimonadetes bacterium KS2-K035	4124119	1196	4612	70.021	4896	113.4x	78.37	2.2	JAABZY000000000	SAMN13684309
Gemmatimonadetes bacterium CS2-K037	Gemmatimonadetes bacterium CS2-K037	3736418	1005	4889	70.369	4386	125.1x	72.15	3.45	JAABXM000000000	SAMN13684245
Gemmatimonadetes bacterium CS4-K037	Gemmatimonadetes bacterium CS4-K037	3704092	985	4922	70.462	4321	126.2x	72.15	0	JAABYU000000000	SAMN13684279
Candidatus Dadabacteria bacterium CS3-K038	Candidatus Dadabacteria bacterium CS3-K038	2213788	239	121452	38.764	2744	211.2x	75.21	1.1	JAABYA000000000	SAMN13684259
Gemmatimonadetes bacterium CS1-K038	Gemmatimonadetes bacterium CS1-K038	4330175	1316	4430	69.68	5228	108x	80.45	2.75	JAABWZ000000000	SAMN13684232
Desulfuromonadales bacterium KS2-K039	Desulfuromonadales bacterium KS2-K039	4591830	1518	4777	58.934	6773	101.8x	82.67	30.7	JAABZZ000000000	SAMN13684310
Gammaproteobacteria bacterium A-K039	Gammaproteobacteria bacterium A-K039	1583786	168	20716	65.84	1695	295.2x	68.67	6.9	JAABWG000000000	SAMN13684213
Gammaproteobacteria bacterium CS4-K039	Gammaproteobacteria bacterium CS4-K039	5940117	2611	2631	67.019	9007	78.7x	60.96	10.75	JAABYV000000000	SAMN13684280
Gemmatimonadetes bacterium CS3-K039	Gemmatimonadetes bacterium CS3-K039	3721095	354	96024	65.911	3726	125.6x	74.14	6.03	JAABYB000000000	SAMN13684260
Gammaproteobacteria bacterium KS1-K040	Gammaproteobacteria bacterium KS1-K040	2502581	282	249877	47.784	2925	186.8x	95.57	7.22	JAABZE000000000	SAMN13684289
Gemmatimonadetes bacterium A-K042	Gemmatimonadetes bacterium A-K042	4144056	1270	4851	69.688	5023	112.8x	67.55	0	JAABWH000000000	SAMN13684214
Gemmatimonadetes bacterium CS3-K043	Gemmatimonadetes bacterium CS3-K043	4268310	1284	4451	69.735	5149	109.5x	77.27	3.3	JAABYC000000000	SAMN13684261
Actinobacteria bacterium A-K045	Microtrichales bacterium A-K045	4761647	2497	16116	65.663	8339	98.2x	70.42	14.1	JAABWI000000000	SAMN13684215
Gammaproteobacteria bacterium KS2-K045	Gammaproteobacteria bacterium KS2-K045	3442481	1139	5168	66.551	4787	135.8x	71.04	12.43	JAACAA000000000	SAMN13684311
Nitrospinaceae bacterium CS1-K045	Nitrospinaceae bacterium CS1-K045	4150208	1211	4917	53.864	5927	112.6x	86.21	32.54	JAABXA000000000	SAMN13684233
Gammaproteobacteria bacterium CS2-K046	Gammaproteobacteria bacterium CS2-K046	3959467	448	60864	65.622	4345	118.1x	64.66	6.72	JAABXN000000000	SAMN13684246
Phycisphaerae bacterium KS1-K047	Phycisphaerae bacterium KS1-K047	6226245	853	21082	46.97	6526	75.1x	88.07	19.52	JAABZF000000000	SAMN13684290
Gemmatimonadetes bacterium A-K048	Gemmatimonadetes bacterium A-K048	4118841	475	86694	65.582	4277	113.5x	91.14	13.32	JAABWJ000000000	SAMN13684216
Gemmatimonadetes bacterium KS1-K048	Gemmatimonadetes bacterium KS1-K048	4323138	1319	24656	69.675	5238	108.1x	78.37	2.75	JAABZG000000000	SAMN13684291
Gammaproteobacteria bacterium KS2-K049	Gammaproteobacteria bacterium KS2-K049	2662553	554	20523	65.129	3237	175.6x	70.95	6.53	JAACAB000000000	SAMN13684312
Gammaproteobacteria bacterium A-K050	Gammaproteobacteria bacterium A-K050	2342298	163	65066	65.334	2399	199.6x	79.36	8.63	JAABWK000000000	SAMN13684217
Actinobacteria bacterium CS1-K051	Microtichales bacterium CS1-K051	5194371	2724	1914	65.789	8954	90x	66.84	11.54	JAABXB000000000	SAMN13684234
Gemmatimonadetes bacterium ACK-K052	Gemmatimonadetes bacterium ACK-K052	3662107	995	4494	70.354	4277	127.7x	68.33	3.85	JAABVT000000000	SAMN13684200
Gemmatimonadetes bacterium KS1-K052	Gemmatimonadetes bacterium KS1-K052	4268477	524	86694	65.529	4460	109.5x	91.14	8.79	JAABZH000000000	SAMN13684292
Gemmatimonadetes bacterium KS4-K052	Gemmatimonadetes bacterium KS4-K052	4249362	1318	4393	69.597	5149	110x	71.63	2.75	JAACBI000000000	SAMN13684345
Actinobacteria bacterium KS2-K053	Microtrichales bacterium KS2-K053	5787870	3054	1883	65.472	9948	80.8x	74.36	17.97	JAACAC000000000	SAMN13684313
Gammaproteobacteria bacterium CS3-K053	Gammaproteobacteria bacterium CS3-K053	3952722	341	65644	65.165	4244	118.3x	64.66	6.4	JAABYD000000000	SAMN13684262
Gammaproteobacteria bacterium KS1-K053	Gammaproteobacteria bacterium KS1-K053	5441380	481	60313	65.857	5781	85.9x	75.86	22.1	JAABZI000000000	SAMN13684293
Nitrospinaceae bacterium CS4-K053	Nitrospinaceae bacterium CS4-K053	4177482	1224	4834	53.893	5951	111.9x	86.05	30.99	JAABYW000000000	SAMN13684281
Actinobacteria bacterium CS4-K054	Microtrichales bacterium CS4-K054	5121017	2671	16116	65.766	8800	91.3x	69.61	15.03	JAABYX000000000	SAMN13684282
Gemmatimonadetes bacterium KS1-K054	Gemmatimonadetes bacterium KS1-K054	5800070	2550	2666	67.108	8840	80.6x	62.11	8.49	JAABZJ000000000	SAMN13684294
Candidatus Saccharibacteria bacterium CS1-K056	Patescibacteria bacterium CS1-K056	2372810	1064	32014	41.527	4182	197x	83.62	5.54	JAABXC000000000	SAMN13684235
Gammaproteobacteria bacterium KS3-K056	Gammaproteobacteria bacterium KS3-K056	3372302	381	86349	63.685	3805	138.6x	71.55	11.82	JAACAT000000000	SAMN13684330
Gammaproteobacteria bacterium CS3-K057	Gammaproteobacteria bacterium CS3-K057	2630578	593	18789	64.892	3270	177.7x	68.78	6.61	JAABYE000000000	SAMN13684263
Actinobacteria bacterium KS3-K058	Microtrichales bacterium KS3-K058	4955341	2550	16116	65.526	8490	94.3x	69.57	13.53	JAACAU000000000	SAMN13684331
Desulfobacterales bacterium KS1-K059	Desulfatiglandales bacterium KS1-K059	4331908	2148	2121	46.545	8281	107.9x	59.73	8.21	JAABZK000000000	SAMN13684295
Desulfuromonadales bacterium KS1-K060	Desulfuromonadales bacterium KS1-K060	4657812	1722	3715	57.952	7277	100.4x	79.87	27.12	JAABZL000000000	SAMN13684296
Gammaproteobacteria bacterium KS3-K060	Gammaproteobacteria bacterium KS3-K060	3973620	1147	15662	46.539	5889	117.7x	96.62	21.74	JAACAV000000000	SAMN13684332
Gemmatimonadetes bacterium ACK-K060	Gemmatimonadetes bacterium ACK-K060	4115591	460	86694	65.658	4252	113.6x	91.14	9.09	JAABVU000000000	SAMN13684201
Gemmatimonadetes bacterium KS4-K060	Gemmatimonadetes bacterium KS4-K060	4115591	460	86694	65.658	4252	113.6x	91.14	13.32	JAACBJ000000000	SAMN13684346
Gammaproteobacteria bacterium KS2-K061	Gammaproteobacteria bacterium KS2-K061	4711840	364	65644	66.024	4945	99.2x	76.72	20.38	JAACAD000000000	SAMN13684314
Candidate division Zixibacteria bacterium CS1-K062	Candidate division Zixibacteria bacterium CS1-K062	4068351	912	9900	45.667	5163	114.9x	87.36	6.04	JAABXD000000000	SAMN13684236
Candidate division KSB1 bacterium CS1-K065	Candidate division KSB1 bacterium CS1-K065	6581933	951	19754	46.846	7919	71x	83.94	8.28	JAABXE000000000	SAMN13684237
Phycisphaerae bacterium ACK-K067	Phycisphaerae bacterium ACK-K067	7301894	1352	17406	46.419	8721	64x	96.49	40.88	JAABVV000000000	SAMN13684202
Phycisphaerae KS4-K067	Phycisphaerae KS4-K067	7301894	1352	19659	46.419	8720	64x	84.66	8.89	JAACBK000000000	SAMN13684347
Gammaproteobacteria bacterium ACK-K068	Gammaproteobacteria bacterium ACK-K068	2574807	522	74003	64.854	3099	181.6x	86.21	6.9	JAABVW000000000	SAMN13684203
Gammaproteobacteria bacterium KS4-K068	Gammaproteobacteria bacterium KS4-K068	2574807	522	20716	64.854	3099	181.6x	68.67	6.9	JAACBL000000000	SAMN13684348
Candidate division KSB1 bacterium CS4-K069	Candidate division KSB1 bacterium CS4-K069	6523522	925	20087	46.841	7847	71.7x	86.13	9.41	JAABYZ000000000	SAMN13684284
Phycisphaerae bacterium CS3-K069	Phycisphaerae bacterium CS3-K069	3977641	451	22113	46.957	4109	117.5x	56.25	1.24	JAABYF000000000	SAMN13684264
Gammaproteobacteria bacterium ACK-K070	Gammaproteobacteria bacterium ACK-K070	4761534	411	102188	65.618	5089	98.2x	100	4.67	JAABVX000000000	SAMN13684204
Gammaproteobacteria bacterium KS4-K070	Gammaproteobacteria bacterium KS4-K070	4761534	411	65066	65.618	5089	98.2x	79.36	8.63	JAACBM000000000	SAMN13684349
Nitrospinaceae bacterium KS1-K070	Nitrospinaceae bacterium KS1-K070	4257554	1268	4568	54.101	6077	109.8x	86.21	32.54	JAABZM000000000	SAMN13684297
Nitrospinaceae bacterium CS3-K072	Nitrospinaceae bacterium CS3-K072	4287540	1281	4563	54.067	6141	109x	87.93	31.68	JAABYH000000000	SAMN13684266
Nitrospinaceae bacterium CS2-K074	Nitrospinaceae bacterium CS2-K074	4140984	1203	4938	53.891	5906	112.9x	87.93	29.8	JAABXO000000000	SAMN13684247
Nitrospinaceae bacterium KS2-K074	Nitrospinaceae bacterium KS2-K074	4234516	1231	4986	53.805	6046	110.4x	89.66	32.54	JAACAE000000000	SAMN13684315
Candidate division Zixibacteria bacterium CS4-K075	Candidate division Zixibacteria bacterium CS4-K075	4698182	1244	7051	45.1	6402	99.5x	79.67	12.45	JAABZA000000000	SAMN13684285
Candidate division Zixibacteria bacterium ACK-K077	Candidate division Zixibacteria bacterium ACK-K077	2661730	371	7152	46.409	3101	175.6x	87.91	10.44	JAABVY000000000	SAMN13684205
Candidate division Zixibacteria bacterium KS4-K077	Candidate division Zixibacteria bacterium KS4-K077	3311482	681	10712	45.963	4142	141.2x	81.87	4.76	JAACBN000000000	SAMN13684350
Candidatus Dadabacteria bacterium KS2-K077	Candidatus Dadabacteria bacterium KS2-K077	2823570	400	131317	39.225	3537	165.6x	97.74	3.3	JAACAF000000000	SAMN13684316
Akkermansiaceae bacterium KS3-K078	Akkermansiaceae bacterium KS3-K078	5426097	2380	2576	60.508	8657	86.2x	63.24	4.56	JAACAW000000000	SAMN13684333
Nitrospinaceae bacterium A-K078	Nitrospinaceae bacterium A-K078	4258367	1247	4282	53.788	6083	109.8x	91.38	31.52	JAABWL000000000	SAMN13684218
Anaerolineae bacterium CS3-K079	Anaerolineae bacterium CS3-K079	9418725	3981	2751	58.652	15109	49.6x	76.93	26.32	JAABYI000000000	SAMN13684267
Gammaproteobacteria bacterium CS4-K079	Gammaproteobacteria bacterium CS4-K079	2125293	329	360840	48.197	2740	220x	75.57	4.35	JAABZB000000000	SAMN13684286
Gammaproteobacteria bacterium KS2-K079	Gammaproteobacteria bacterium KS2-K079	3762580	1075	54292	46.005	5492	124.2x	95.92	13.86	JAACAG000000000	SAMN13684317
Candidate division KSB1 bacterium KS1-K080	Candidate division KSB1 bacterium KS1-K080	6248850	888	20272	46.8	7531	74.8x	83.94	9.28	JAABZO000000000	SAMN13684299
Candidatus Saccharibacteria bacterium KS3-K080	Patescibacteria bacterium KS3-K080	763255	242	32014	41.856	1227	612.5x	83.62	5.54	JAACAX000000000	SAMN13684334
Gammaproteobacteria bacterium CS1-K080	Gammaproteobacteria bacterium CS1-K080	2213352	603	5000	60.189	3098	211.2x	56.9	4.86	JAABXF000000000	SAMN13684238
Nitrospinaceae bacterium ACK-K080	Nitrospinaceae bacterium ACK-K080	4757634	1499	4917	53.633	7007	98.3x	89.66	31.68	JAABVZ000000000	SAMN13684206
Nitrospinaceae bacterium KS4-K080	Nitrospinaceae bacterium KS4-K080	4336596	1292	4768	53.853	6240	107.8x	89.66	29.8	JAACBO000000000	SAMN13684351
Candidatus Dadabacteria bacterium A-K082	Candidatus Dadabacteria bacterium A-K082	2137304	261	13582	37.997	2665	218.7x	88.61	36.52	JAABWM000000000	SAMN13684219
Candidatus Saccharibacteria bacterium KS1-K082	Patescibacteria bacterium KS1-K082	1204052	427	23171	41.392	1993	388.3x	85.87	8.46	JAABZP000000000	SAMN13684300
Gammaproteobacteria bacterium CS1-K082	Gammaproteobacteria bacterium CS1-K082	6377343	2006	4459	45.276	9493	73.3x	95.35	27.28	JAABXG000000000	SAMN13684239
Phycisphaerae bacterium KS3-K082	Phycisphaerae bacterium KS3-K082	6826643	1175	21521	46.173	7925	68.5x	93.75	10.27	JAACAY000000000	SAMN13684335
Calditrichae bacterium CS3-K084	Calditrichia bacterium CS3-K084	1250942	430	4202	42.235	2016	373.7x	69.87	18.34	JAABYJ000000000	SAMN13684268
Phycisphaerae bacterium A-K084	Phycisphaerae bacterium A-K084	6851300	1235	17867	46.182	7891	68.2x	63.16	9.01	JAABWO000000000	SAMN13684221
Candidate division KSB1 bacterium CS3-K085	Candidate division KSB1 bacterium CS3-K085	6034281	712	21073	46.992	7027	77.5x	83.94	5.98	JAABYK000000000	SAMN13684269
Candidate division Zixibacteria bacterium A-K086	Candidate division Zixibacteria bacterium A-K086	5221349	1378	13659	45.368	6898	89.5x	74.11	0	JAABWP000000000	SAMN13684222
Candidate division Zixibacteria bacterium KS1-K086	Candidate division Zixibacteria bacterium KS1-K086	4601281	1239	6786	45.078	6310	101.6x	79.67	13	JAABZQ000000000	SAMN13684301
Candidate division KSB1 bacterium A-K087	Candidate division KSB1 bacterium A-K087	6646535	937	20570	46.911	7930	70.3x	83.94	4.69	JAABWQ000000000	SAMN13684223
Candidate division Zixibacteria bacterium CS3-K087	Candidate division Zixibacteria bacterium CS3-K087	4675434	1261	6980	45.238	6340	100x	79.67	13	JAABYL000000000	SAMN13684270
Candidate division KSB1 bacterium ACK-K089	Candidate division KSB1 bacterium ACK-K089	6337027	811	20087	46.966	7460	73.8x	85.04	21.17	JAABWA000000000	SAMN13684207
Candidate division KSB1 bacterium KS4-K089	Candidate division KSB1 bacterium KS4-K089	6337027	811	20570	46.966	7460	73.8x	83.94	4.69	JAACBP000000000	SAMN13684352
Calditrichae bacterium A-K090	Calditrichia bacterium A-K090	2403277	895	3341	41.499	3951	194.5x	76.54	25.94	JAABWR000000000	SAMN13684224
Candidate division KSB1 bacterium KS2-K090	Candidate division KSB1 bacterium KS2-K090	6602385	991	19584	46.728	8010	70.8x	83.94	10.38	JAACAH000000000	SAMN13684318
Aliifodinibius sp. CS3-K091	Aliifodinibius sp. CS3-K091	6018302	2294	3238	40.379	9494	77.7x	71.69	20.67	JAABYM000000000	SAMN13684271
Phycisphaerae bacterium CS2-K091	Phycisphaerae bacterium CS2-K091	2909774	407	21133	46.688	3296	160.7x	87.62	27.65	JAABXR000000000	SAMN13684250
Candidate division KSB1 bacterium CS2-K093	Candidate division KSB1 bacterium CS2-K093	6628716	1019	19294	46.603	8184	70.5x	91.08	12.91	JAABXS000000000	SAMN13684251
Aliifodinibius sp. ACK-K096	Aliifodinibius sp. ACK-K096	6397534	2531	3032	40.969	10275	73.1x	72.29	20.9	JAABWB000000000	SAMN13684208
Aliifodinibius sp. KS4-K096	Aliifodinibius sp. KS4-K096	6397534	2531	3032	40.969	10275	73.1x	72.29	20.9	JAACBR000000000	SAMN13684354
Calditrichae bacterium CS2-K097	Calditrichia bacterium CS2-K097	1664086	578	3919	42.188	2722	280.9x	68.62	17.32	JAABXU000000000	SAMN13684253
Candidate division Zixibacteria bacterium KS2-K099	Candidate division Zixibacteria bacterium KS2-K099	6006093	1373	9381	45.507	7654	77.8x	80.77	19.23	JAACAI000000000	SAMN13684319
Candidate division Zixibacteria bacterium KS3-K099	Candidate division Zixibacteria bacterium KS3-K099	3538399	727	9947	45.622	4473	132.1x	79.67	8.06	JAACBA000000000	SAMN13684337
