# Author Correction: 309 metagenome assembled microbial genomes from deep sediment samples in the Gulfs of Kathiawar Peninsula

**DOI:** 10.1038/s41597-021-01027-1

**Published:** 2021-09-17

**Authors:** Neelam M. Nathani, Kaushambee J. Dave, Priyanka P. Vatsa, Mayur S. Mahajan, Parth Sharma, Chandrashekar Mootapally

**Affiliations:** 1 Department of Life Sciences, Maharaja Krishnakumarsinhji Bhavnagar University, Bhavnagar, 364001 Gujarat India; 2 Department of Molecular Cell Biology and Immunology, University of Tübingen, Geschwister-Scholl-Platz, 72074 Tübingen, Germany; 3 Department of Biotechnology, National Institute of Pharmaceutical Education and Research (NIPER), Gandhinagar, 382355 Gujarat India; 4 Microbiology Division, Regional Centre, Lokhandwala Road, Four Bungalows, Andheri (West), CSIR – National Institute of Oceanography (CSIR-NIO), Mumbai, 400053 Maharashtra India; 5 Department of Marine Science, Maharaja Krishnakumarsinhji Bhavnagar University, Bhavnagar, 364001 Gujarat India

**Keywords:** Environmental biotechnology, Biodiversity

Correction to: *Scientific Data* 10.1038/s41597-021-00957-0, published online 28 July 2021

The original version of this Data Descriptor contained an error in the legend of Figure 2, which incorrectly stated that five MAGs were classified, this has been corrected to four. In the same figure, the proteobacteria phyla count for GOC has been corrected to 56 from 57. These errors have now been corrected in both the PDF and HTML versions of the Data Descriptor.

